# Notes and News

**Published:** 1904-09-10

**Authors:** 


### Notes and News.

The King has sent his annual subscription of £105 to his Hospital Fund for London.

At the St. Louis Exhibition the Johns Hopkins Hospital, Baltimore, has a special display. The complete work within the hospital is shown by means of photographs, models, and specimens.

A new isolation hospital has been opened for the Upton Rural District. At present there are three blocks of buildings. The ward blocks contain 10 beds each, and the administrative block is occupied by the usual offices, surgery, and six bedrooms; the laundry block contains also disinfecting rooms, mortuary, and other offices. The architect is Mr. Lewis Sheppard, of Worcester, and the cost was £5,000.

At the Bideford Hospital a charge of one shilling is made to each patient. It was suggested at the annual meeting of the North Devon Infirmary that this procedure should be adopted. We have always maintained that a payment according to means should be made by every patient not being a pauper. But the arbitrary charge of one shilling does not appeal to our judgment. The result is very small, it may be regarded as a grievance by some very poor patients and as an absurdity by the well-to-do, and is likely to check the flow of spontaneous gratitude which should be encouraged from those who enjoy the benefits of the hospitals.

The definition as to what actually constitutes a conscientious objection has obviously admitted of many interpretations up to the present time. Now the Home Office has issued a statement which should render the magistrates' task much easier. The Lord Chief Justice some little time back defined the grounds upon which a man may be regarded as a conscientious objector. He said that it is not sufficient for a man to object to vaccination on general grounds, but he must be prepared to maintain that vaccination in his belief would be prejudicial in the case of a particular child. This is the course which the Home Office wishes to be generally followed.

The *Maine* Hospital Ship has gone to Smyrna.

Mr. Carnegie has promised £1,000 towards the Darlington Hospital Extension Fund.

The late Mrs. E. M. Girling of Colwyn Bay has bequeathed the ultimate residue of her property to the Stockport Infirmary.

Sir Riley Lord will present a statue of Queen Victoria to the Newcastle Infirmary. It will be placed in the grounds of the new infirmary.

A Royal Commission has been appointed to consider the existing methods of dealing with idiots and epileptics and the feeble-minded and defective, and to advise on the subject. The Commissioners are the Marquis of Bath, W. Patrick Byrne, C.B., Charles Hobhouse, M.P., Frederick Needham, M.D., Henry David Greene, K.C., M.P., Charles E. Heley Chadwyck Healey, Rev. H. N. Burden, Willoughby H. Dickinson, K.C., C. S. Loch, Esq., and Mrs. Pinset.

We were recently asked by a military man if any technical qualifications were necessary for the post of a hospital secretary. He went on to add that he supposed not, as a friend had applied for such a position, and beyond considerable social influence he had no knowledge outside military matters, and certainly he knew nothing about hospitals, their finances, or management. Since these inquiries were made, an extract from the rules of the Great Ormond Street Children's Hospital, which is seeking a secretary, has come into our hands, and supplies an example of the varied requirements of such an office. These rules have been issued to candidates. It is very obvious that the position so ably filled by the late Mr. Adrian Hope demands no small knowledge and no mean abilities. In brief, the secretary is required to supervise the upkeep and repairs of the buildings, be responsible for the discipline of the male domestic staff; he must keep all accounts and prepare all documents put before the committees, and collect money and improve the income of the hospital. For the satisfactory performance of these duties the salary of £400 per annum is offered. It will be seen how very various and technical are the requirements of the office. In the instructions to candidates we note that the committee emphasise the importance they attach to the power of the secretary to obtain financial support for the hospital. It is seldom an easy task to find the right man for any post, but to find a good hospital secretary must be exceptionally difficult. Obviously it is required that the candidate in this case should be of good social standing. But a person of good social standing of not more than 45 years of age, who is also a man of ability and possessing an incentive to work who is content with a salary of £400 per annum with office hours from 10 to 5, is not easy to find. The amount of administrative work required demands experienced ability, whilst the collector's duties require personal qualities and powers of an equally high order in another direction. It was this unusual combination of qualities which rendered Mr. Adrian Hope so valuable an officer.

